# The Grapevine E3 Ubiquitin Ligase VriATL156 Confers Resistance against the Downy Mildew Pathogen *Plasmopara viticola*

**DOI:** 10.3390/ijms22020940

**Published:** 2021-01-19

**Authors:** Elodie Vandelle, Pietro Ariani, Alice Regaiolo, Davide Danzi, Arianna Lovato, Claudia Zadra, Nicola Vitulo, Giorgio Gambino, Annalisa Polverari

**Affiliations:** 1 Department of Biotechnology, University of Verona, Strada Le Grazie 15, CV1, 37134 Verona, Italy; pietro.ariani@gmail.com (P.A.); aregaiol@uni-mainz.de (A.R.); davide.danzi@univr.it (D.D.); ariannahim@libero.it (A.L.); nicola.vitulo@univr.it (N.V.); 2 Department of Pharmaceutical Sciences, University of Perugia, Borgo XX Giugno 72, 06121 Perugia, Italy; claudia.zadra@unipg.it; 3 Institute for Sustainable Plant Protection, National Research Council (IPSP-CNR), Strada delle Cacce 73, 10135 Torino, Italy; giorgio.gambino@ipsp.cnr.it

**Keywords:** ATL E3 ubiquitin ligases, plant functional genomics, promoter analysis, plant resistance, sustainable agriculture

## Abstract

Downy mildew, caused by *Plasmopara viticola*, is one of the most severe diseases of grapevine (*Vitis vinifera* L.). Genetic resistance is an effective and sustainable control strategy, but major resistance genes (encoding receptors for specific pathogen effectors) introgressed from wild *Vitis* species, although effective, may be non-durable because the pathogen can evolve to avoid specific recognition. Previous transcriptomic studies in the resistant species *Vitis riparia* highlighted the activation of signal transduction components during infection. The transfer of such components to *V. vinifera* might confer less specific and therefore more durable resistance. Here, we describe the generation of transgenic *V. vinifera* lines constitutively expressing the *V. riparia* E3 ubiquitin ligase gene *VriATL156*. Phenotypic and molecular analysis revealed that the transgenic plants were less susceptible to *P. viticola* than vector-only controls, confirming the role of this E3 ubiquitin ligase in the innate immune response. Two independent transgenic lines were selected for detailed analysis of the resistance phenotype by RNA-Seq and microscopy, revealing the profound reprogramming of transcription to achieve resistance that operates from the earliest stages of pathogen infection. The introduction of *VriATL156* into elite grapevine cultivars could therefore provide an effective and sustainable control measure against downy mildew.

## 1. Introduction

Disease resistance in plants is controlled by multiple genetic and epigenetic mechanisms, including general physical barriers and chemical defense compounds as well as the recognition of pathogen-associated molecular patterns (PAMPs) or race-specific pathogen effectors via pattern recognition receptors (PRRs) or receptors encoded by resistance (*R*) genes, respectively. Signaling via such receptors results in the transcriptional and metabolic reprogramming of plant cells to improve defense and prevent colonization by pathogens [1]. The genes and metabolites involved in resistance have been determined in model plant species, including key signal transduction components and metabolic pathways that produce antimicrobial compounds. This knowledge can now be used to generate disease-resistant varieties of important crops such as grapevine (*Vitis vinifera* L.), facilitated by the availability of genetic and genomic resources such as the complete genome sequence, gene expression databases, gene family catalogs, and transformation protocols [2,3].

Grapevine is susceptible to many pathogens, but one of the most economically important is the oomycete *Plasmopara viticola* (Berk. et Curt.) Berl. & de Toni, which is responsible for downy mildew. This devastating disease can only be controlled by applying multiple pesticide sprays per season, and downy mildew resistance is therefore one of the most desired traits in grapevine breeding. Genetically inherited resistance would help to reduce the environmental and economic costs of current disease control methods. This has been achieved by crossing with wild *Vitis* relatives in a huge breeding effort that began in the last century [4,5]. Several resistance genes have been mapped in segregating progeny, facilitating marker assisted selection to speed up the breeding process. The molecular bases of the resistance mechanisms are not fully understood, but appear to involve the post-infection activation of a hypersensitive response and the accumulation of reactive oxygen species (ROS) as well as antimicrobial proteins and secondary metabolites [6]. A very recent proteomic analyses showed that a plastic plant response is already active 12 h post-infection in the highly tolerant cv. Regent [7].

Despite the development of several new *V. vinifera* cultivars with resistance against *P. viticola* as well as desirable organoleptic properties [8,9], the wine industry still relies on traditional tastes and brands that cannot be maintained following the crossing and modification of the corresponding genetic assets. Genetic engineering allows the introduction of resistance traits directly into elite cultivars without altering the genetic background, maintaining the taste and aroma of the resulting wines. However, only a few examples of disease-resistant transgenic grapevines have been reported, mainly due to technical barriers such as the recalcitrance of many cultivars to gene transfer and regeneration, which prevents the development of transgenic lines and the functional analysis of candidate genes by transient expression [10]. Several *R* gene analogues (RGAs) have been identified in wild grapevine relatives such as *Muscadinia rotundifolia* (https://www.vivc.de/index.php), but only two have been cloned and transferred to *V. vinifera* thus far: *MrRpv1* and *MrRun1*, conferring resistance to downy and powdery mildew isolates, respectively [11]. Many more RGAs used in classical breeding programs await isolation, cloning and transfer to cultivated grapevine varieties. However, the lack of information about the elicitors detected by these RGAs poses a risk that pathogens could rapidly evolve to evade detection [12]. In contrast, targeting the downstream signaling components would allow the development of transgenic plants with less specific and therefore more durable resistance. Among the examples of such components, the constitutive or ectopic overexpression of defense-related WRKY transcription factors in *V. vinifera* has reduced susceptibility to *P. viticola* compared to wild-type control plants [13,14]. Key signaling components could also be introduced into a susceptible genotype by a cisgenic approach (i.e., the transfer of genes in their native form, including promoter and regulatory regions from sexually compatible species). However, the implementation of such an approach requires further detailed analysis of gene promoters to ensure that the introduced gene is expressed in the optimal manner to achieve the desired level of resistance. In turn, the careful investigation of promoter responses also provides the basis for the engineering of synthetic promoters [15,16].

We previously described a small group of grapevine genes encoding RING-H2 E3 ubiquitin ligases that were more strongly or specifically expressed in the resistant species *Vitis riparia* than its susceptible relative *V. vinifera* following infection with *P. viticola* [17]. All these genes belong to the ATL subfamily, associated with defense in *Arabidopsis thaliana* and other species [18,19,20,21,22]. Following extensive characterization of the grapevine ATL gene family and meta-analysis of the transcriptome datasets, we found that the expression of these genes is induced in response to a range of pathogens [20] and co-regulated together with key components of the resistance signaling cascade [22]. We therefore selected the *V. riparia* ortholog of the *VviATL156* gene (tentatively named *VriATL156*) for further investigation because it was the most strongly and rapidly induced in *V. riparia* as early as 12 h post-infection (hpi) with *P. viticola*. The differential expression of the *V. riparia* and *V. vinifera ATL156* genes was investigated by functional analysis of the promoter regions in silico and in vivo in transgenic *A. thaliana* plants, and the involvement of ATL156 in resistance to downy mildew was studied by the overexpression of *VriATL156* in transgenic *V. vinifera* plants, which reduced their susceptibility to downy mildew and induced a profound transcriptional reprogramming of defense-related genes.

## 2. Results

### 2.1. Bioinformatic Analysis of the V. vinifera and V. riparia ATL156 Genes

*VviATL156* (VIT_05s0077g01970) is the *V. vinifera* ATL gene most closely related to *A. thaliana* defense-related gene *AtATL2* [18,20]. *VviATL156* features a 954-bp coding sequence without introns, flanked by 5′ and 3′ untranslated regions (UTRs) of 98 and 322 bp, respectively. Our previous microarray analysis of grapevine plants infected with *P. viticola* revealed the weak induction of *VviATL156* 24 hpi in *V. vinifera*, whereas the *V. riparia* ortholog was induced earlier (12 hpi) and more strongly [17].

The differential response of the *ATL156* gene in *V. vinifera* and *V. riparia* was initially investigated in silico by comparing the coding sequences and promoters (Supplementary Figures S1 and S2). Due to the lack of the *V. riparia* genome at that time, the *VriATL156* gene was amplified from *V. riparia* cv. Gloire de Montpellier genomic DNA using primers designed for the corresponding *V. vinifera* gene. The resulting amplicon was 960 bp in length, indicating that *VriATL156* is intronless like its ortholog in *V. vinifera* and most other ATL genes [20,23]. Comparison of the *VviATL156* and the *VriATL156* coding sequences revealed very high similarity, with only five single nucleotide polymorphisms (SNPs) and one additional tandem duplication of a 6-bp sequence that is actually a tandem repeat in *V. vinifera* and a triplet repeat in *V. riparia* (Supplementary Figure S1a). At the protein level, three of the five SNPs were silent and the other two resulted in changes from Asp (D) to Glu (E) at position 246 and from Thr (T) to Pro (P) at position 309 in the *V. riparia* polypeptide. The 6-bp tandem duplication caused the insertion of Pro (P) and Gln (Q) at positions 313 and 314, respectively, in the *V. riparia* polypeptide (Supplementary Figure S1b).

The 1174-bp *VviATL156* promoter sequence was cloned for re-sequencing, allowing us to design primers to clone a corresponding 1186-bp *VriATL156* promoter fragment. Sequence analysis showed that the two promoter regions shared 96% identity (Supplementary Figure S2). The prediction of promoter motifs in silico using PlantCARE [24] also revealed a near identical distribution and frequency of *cis*-regulatory elements between the two promoters, including the presence of two TATA boxes (Supplementary Figure S3a). Neither of the promoters featured pathogenesis-related *cis*-regulatory elements, but both contained hormone-response motifs, particularly for salicylic acid [25] and jasmonic acid [26]. Many of the predicted motifs have been associated with transcription factors involved in stress responses. The most abundant motifs in both promoters were those recognized by basic leucine zipper (bZIP) transcription factors, which are known to regulate stress and defense responses and well as light-dependent signaling. Binding sites for WRKY and MYB transcription factors were also abundant in both promoters [27]. Other motifs were identified as binding sites for basic helix loop helix (bHLH), DNA-binding with one finger (Dof) and AT-Hook family transcription factors, which are not specific for defense or stress signaling but regulate developmental processes (Supplementary Figure S3b). The high level of similarity between the promoters did not reveal any clear distinctions that would explain the differential regulation of the *V. vinifera* and *V. riparia ATL156* genes, so we decided to test their functions directly in *A. thaliana*.

### 2.2. The V. riparia ATL156 Promoter is More Responsive to Pathogens and Hormone Treatment

The *V. vinifera* and *V. riparia ATL156* promoters were fused to the *gusA* reporter gene, allowing their activity to be compared in transgenic *A. thaliana* plants by analyzing β-glucuronidase (GUS) activity. Two independent lines for each promoter were examined under normal growth conditions throughout the developmental cycle from seedling emergence to flowering. GUS activity was weaker in lines carrying the *V. riparia* promoter than in lines carrying the *V. vinifera* promoter, as shown by the intensity of GUS staining (Supplementary Figure S4a). This was in agreement with the higher basal levels of *ATL156* mRNA detected in *V. vinifera* compared to *V. riparia* (Supplementary Figure S4b) and confirmed the suitability of the heterologous *A. thaliana* system for the analysis of these promoters.

**Figure 1:**
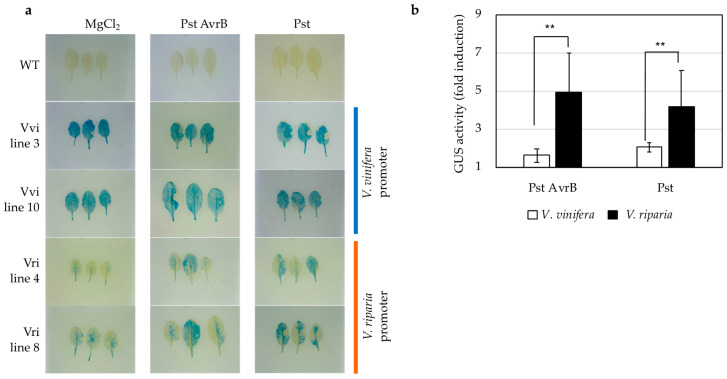
*ATL156* promoters respond differently to pathogen infection. (**a**) β-glucuronidase (GUS) staining and (**b**) fold change in quantified GUS enzymatic activity in *Arabidopsis thaliana* leaves expressing the *gusA* gene under the control of the *Vitis vinifera* or *Vitis riparia ATL156* promoters, 24 h after infection with an avirulent *Pseudomonas syringae* pv. *tomato* (Pst DC3000) strain carrying the avirulence *AvrB* gene (Pst AvrB) or a virulent strain of Pst DC3000. Values are means of three biological replicates ± SE and statistically significant differences calculated with Student’s *t*-test are indicated as **, *p* ≤ 0.01.

To assess the response of the *ATL156* promoters to pathogens, transgenic *A. thaliana* lines expressing each promoter-reporter system were infected with avirulent or virulent strains of *Pseudomonas syringae* pv. *tomato* DC3000 (Pst), the former carrying the avirulence gene *AvrB*. The analysis of GUS activity revealed that both promoters were induced by the pathogen, regardless of the virulence of the strain (Figure 1). However, there was a significant difference in the induction ratio, with the *V. vinifera* promoter triggering a 1.6–2-fold increase in GUS levels and the *V. riparia* promoter triggering a 4–5-fold increase, in both cases relative to a mock infection. Thus, the *V. riparia* promoter clearly showed a stronger response to the pathogen.

**Figure 2:**
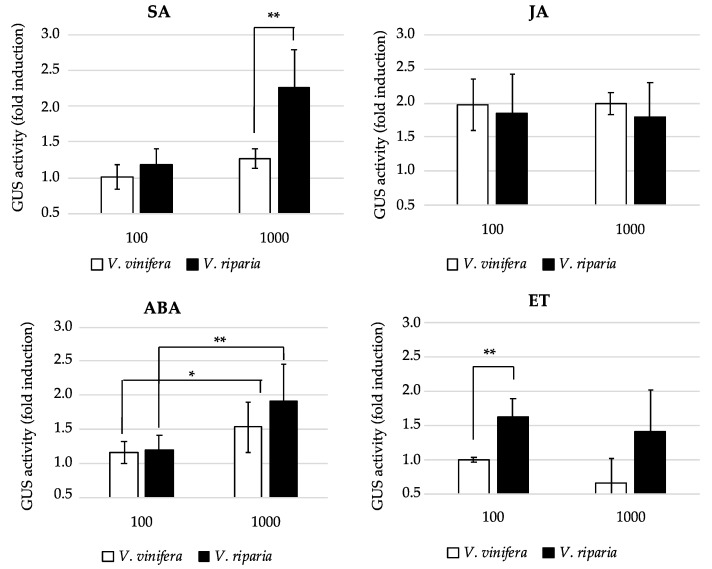
ATL156 promoters respond differently to hormone treatments. GUS activity induction measured in *Arabidopsis thaliana* leaves expressing the *gusA* gene under the control of the *Vitis vinifera* or *Vitis riparia ATL156* promoters, following treatment with salicylic acid (SA), jasmonic acid (JA), abscisic acid (ABA) or the ethylene donor ethephon (ET). Values are means of three biological replicates ± SE and statistically significant differences calculated with Student’s *t*-test are indicated as * = *p* ≤ 0.05; **, *p* ≤ 0.01.

To gain more insight into the molecular basis of this phenomenon, we treated the transgenic plants with various hormones that are known to influence the regulation of ATLs [22]. Both promoters were similarly induced by two different concentrations of jasmonic acid and by 1 mM abscisic acid (ABA), but only the *V. riparia* promoter was induced by 1 mM salicylic acid or 100 µM ethylene (Figure 2). Hormonal regulation may therefore facilitate the stronger response of the *V. riparia* promoter to pathogens.

### 2.3. Transgenic Grapevine Plants Can Express a Functional VriATL156 Gene

We expressed the *VriATL156* coding sequence in *V. vinifera* under the control of the constitutive cauliflower mosaic virus 35S (CaMV 35S) promoter to determine its ability to confer resistance against *P. viticola*. We transformed *V. vinifera* cv. Shiraz callus [28] by co-cultivation with *Agrobacterium tumefaciens* EHA105 carrying the pART27-*VriATL156* binary vector or the empty pART27 vector as a negative control. This vector carries a kanamycin resistance gene and an expression cassette for green fluorescent protein (GFP), allowing dual selection by growth on kanamycin-supplemented medium and visual selection for fluorescence (Supplementary Figure S5a). Southern blot analysis revealed a total of 14 plantlets representing six transformation events, which differ in their number of insertions (Supplementary Figure S5b). All plantlets were regenerated and propagated in vitro (Supplementary Figure S5c) before acclimatization in the greenhouse for another three seasons, except for L7 and L23, which did not survive.

**Figure 3:**
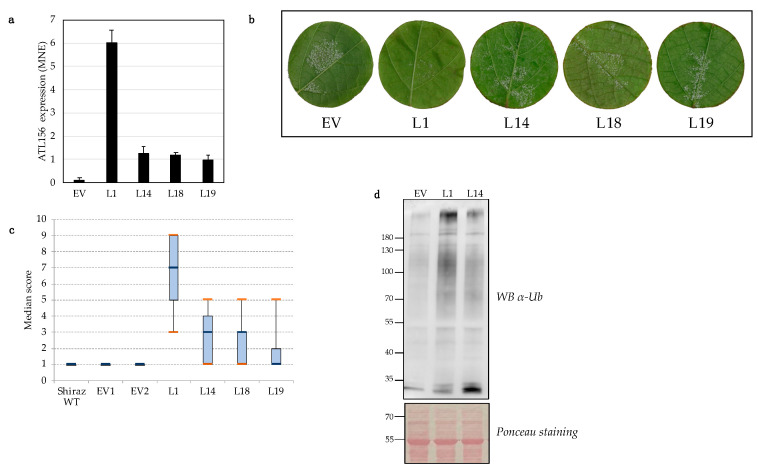
Transgenic *Vitis vinifera* lines express *VriATL156* at different levels, correlating with total ubiquitination levels and resistance to *P. viticola.* (**a**) Expression level of *VriATL156* in different transgenic lines (L1, L14, L18, L19) representing the four remaining transformation events compared to endogenous *VviATL156* expression in the EV (empty vector) control line normalized to the ubiquitin housekeeping gene (VIT_16s0098g01190). Each data point is the mean of three biological replicates ± SE. (**b**) Median resistance score of transgenic (L1, L14, L18, L19) and EV (empty vector) control lines, according to OIV (International Organisation of Vine and Wine) descriptor 452-1, 6 days after inoculation, as estimated from seven independent infection experiments. Orange bars indicate the maximum and minimum scores observed for each plant line. (**c**) Infected leaf disks representative of resistance scores reported in panel (**b**). (**d**) Western blot of total ubiquitinated proteins revealed using a primary anti-ubiquitin antibody 1:2000 and a secondary anti-mouse IgG antibody 1:50,000 in lines L1 and L14 compared to the control line.

*VriATL156* expression was evaluated by qPCR in transgenic lines L1, L14, L18 and L19, representing each of the remaining independent transformation events. The mean normalized expression (MNE) ranged from 1 to 6 depending on the transformation event, compared with a value of 0.13 for the endogenous *VviATL156* gene in control plants. Line L1 showed the highest expression level of *VriATL156*, corresponding to a ~45.5-fold increase in expression compared to *VviATL156* in the controls (Figure 3a).

Functional analysis in four lines, one for each available transformation event, was carried out by infecting leaf disks from the third, fourth and fifth leaves of these lines with *P. viticola*, observed at 6 days post-infection (dpi) for sporulation [29]. The transgenic plants showed enhanced *P. viticola* resistance compared to vector-only controls, correlating with the *VriATL156* expression (Figure 3a–c). Moreover, resistance level correlated with the abundance of total ubiquitinated proteins in transgenic lines L1 and L14, indicating the expression of a functional enzyme (Figure 3d). Infection experiments were carried out at least six times across two consecutive years on at least seven disks per line, with similar sporulation scores.

### 2.4. Resistance to P. viticola in Lines Expressing VriATL156 is Associated with Pathogen Growth Arrest and Enhanced Hormone Production

**Figure 4:**
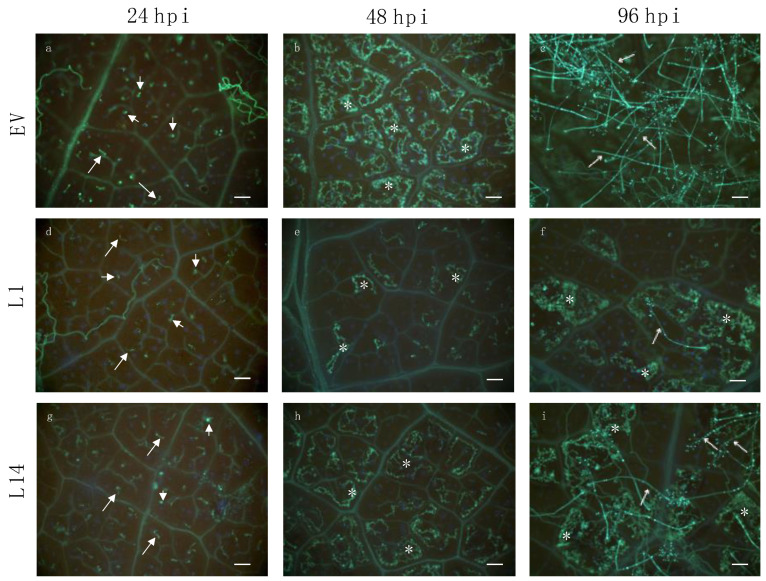
*Plasmopara viticola*-infected leaf disks from *Vitis vinifera* cv. Shiraz plants transformed with the empty vector (EV) or with the *VriATL156* gene under the control of the CaMV 35S promoter (lines L1, L14). Samples were collected at 24, 48 and 96 h post-infection, stained with 0.05% aniline blue and observed by epifluorescence microscopy (magnification = 100×, bars = 100 μm) (**a**–**i**). Short arrows (in (**a**,**d**,**g**)) indicate germinating zoospores; long arrows (in (**a**,**d**,**g**)) indicate germ tubes infecting the leaf through stomata; asterisks indicate patches of irregular mycelium of *P. viticola* developing in the intercellular spaces; thin arrows (in (**c**,**f**,**i**)) indicate branched sporangiophores arising from stomata and bearing brilliant sporangia.

Tissues infected with *P. viticola* were analyzed by microscopy 24, 48 and 96 hpi to visualize the extent of mycelium colonization in the leaves of the two best performing lines (L1 resistant and L14 partially resistant) as well as regenerated, empty vector-transformed plants as controls. The first infection symptoms appeared on control leaves 72 hpi, and sporulation typically occurred 6 dpi. Zoospores were able to germinate at a similar frequency on all plants including controls, but subsequent mycelial growth was severely impaired in line L1, where sporulation was seldom observed (Figure 4), comparable to the outcome of infection in *V. riparia* [17]. We observed no evidence of necrosis or a hypersensitive response related to the infection.

**Figure 5:**
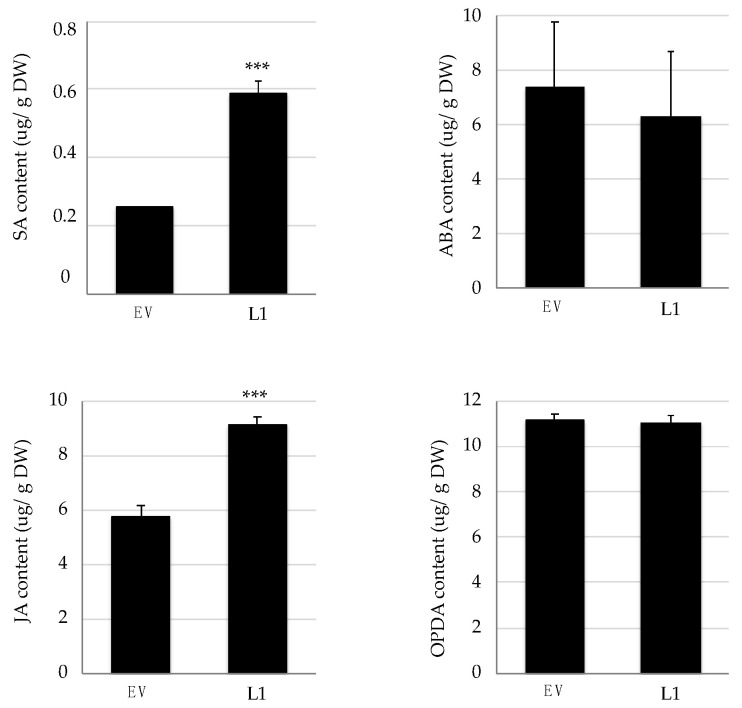
Basal hormone levels in transgenic *Vitis vinifera* plants expressing *VriATL156*. The concentration (expressed in µg/g dry weight) of salicylic acid (SA), jasmonic acid (JA), abscisic acid (ABA) and 12-oxophytodienoic acid (12-OPDA) was measured in the leaves of non-infected control (empty vector) plants and L1 transgenic line expressing *VriATL156* under the control of the CaMV 35S promoter. Data are means of three biological replicates ± SE. Statistically significant differences are indicated as: *** = *p* ≤ 0.01 calculated with Student’s *t*-test.

The measurement of hormone levels revealed that line L1 produced higher basal levels of salicylic acid and jasmonic acid, but the levels of ABA and *cis*-(+)-12-oxophytodienoic acid (OPDA), a precursor of jasmonic acid [30], were unaffected in the absence of infection (Figure 5).

### 2.5. Transcriptional Profiling of Transgenic Grapevine Plants Expressing VriATL156 Indicates the Strong Activation of Defense-Related Signaling before Infection

**Figure 6:**
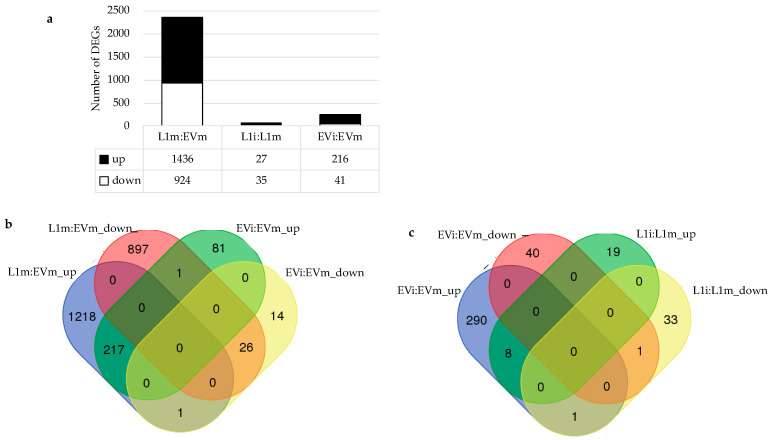
RNA-Seq analysis of transgenic plants expressing *VriATL156.* (**a**) Differentially expressed genes in mock-infected transgenic line L1 (L1m) vs. mock-infected empty-vector control plants (EVm), *P. viticola*-infected L1 (L1i) vs. L1m, and *P. viticola*-infected empty-vector control plants (EVi) vs. EVm. (**b**) Venn diagram showing the overlap between genes constitutively overexpressed in L1 and control plants and the genes induced in control plants by infection with *P. viticola.* (**c**) Venn diagram showing the overlap between genes modulated in infected L1 plants and genes modulated in infected controls. DEGs, differentially expressed genes.

To characterize the molecular phenotype of the transgenic plants expressing *VriATL156*, we compared the most resistant line L1 to vector-only control plants by RNA-Seq analysis before infection with *P. viticola* and 24 hpi. We chose the 24 hpi time point because resistance is fully established 48 hpi, and transcriptional reprogramming is therefore likely to begin at an earlier time (Figure 4). We identified 2360 transcripts that showed significant differential expression (log2FC > |1|, adj. *p*-value < 0.05) in L1 vs. control plants in the absence of infection, 1436 of which were upregulated and 924 downregulated in L1 plants, showing that the constitutive expression of *VriATL156* induces profound transcriptional reprogramming (Figure 6a; Supplementary Dataset S1).

Of note, the expression of *VriATL156* itself was increased (log2FC of 4.3) in transgenic plants, thus confirming the high transcript level of the transgene with this technique. In susceptible control plants, only 257 genes were modulated in response to infection with *P. viticola* (216 upregulated and 41 downregulated) (Figure 6a; Supplementary Dataset S2). Interestingly, around 75% of these genes (192 in total) were also among the 2648 genes modulated constitutively in the non-infected L1 plants, and the directionality of modulation (upregulation or downregulation) was preserved (Figure 6b,c; Supplementary Dataset S3) but the fold difference in expression was much larger in the “L1 uninfected vs. control uninfected” comparison than the “control infected vs. control uninfected” comparison (Supplementary Dataset S3). Finally, the infection of L1 plants caused only minor transcriptional changes, with 27 genes upregulated and 35 downregulated (log2FC > |1|) (Figure 6a; Supplementary Dataset S4). There was very little overlap between the genes induced by infection in the L1 plants and controls (nine genes commonly modulated in both L1i:L1m and EVi:EVm) (Supplementary Dataset S5).

**Figure 7:**
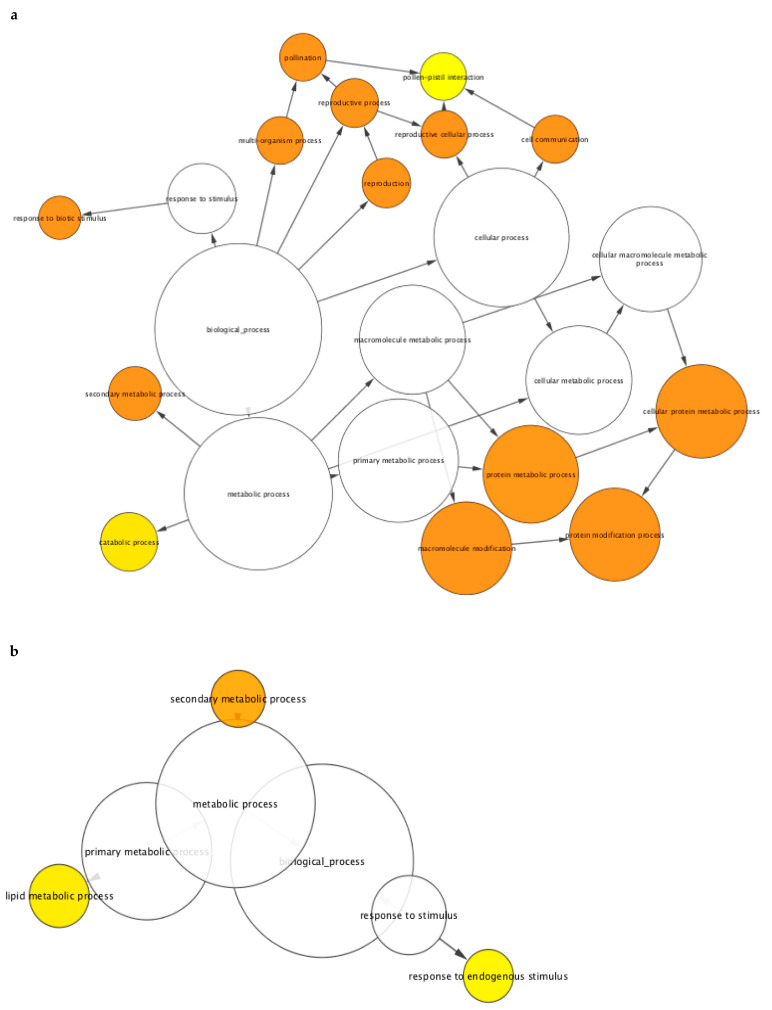
Gene Ontology (GO) functional category enrichment analysis among differentially expressed genes in mock-treated transgenic line L1 (L1m) compared to mock-treated empty-vector control plants (EVm). GO categories of (**a**) upregulated genes and (**b**) downregulated genes significantly enriched were analyzed with BINGO. The size of the nodes is proportional to the number of genes in the test set which are annotated to that node while the color of the node represents the corrected *p*-value. The node color scale ranges from white (not significantly over-represented), yellow (*p*-value < 0.05) to dark orange (*p*-value < 10^−5^ × 0.05).

Enrichment analysis revealed the prevalence of the “protein modification” (265 genes) and “reproduction” (55 genes) functional categories among the genes upregulated in line L1, with both categories mainly comprising receptor kinase-encoding genes (Figure 7a; Supplementary Dataset S6).

In the “protein modification” category, genes encoding mitogen-activated protein kinases (such as MPK3 homologous) and protein phosphatases are also present, as well as numerous proteins involved in protein degradation (such as U-box domain containing proteins). As anticipated, this group included *VriATL156* itself, which was strongly expressed in the L1 plants relative to the endogenous expression of *VviATL156* in the controls (log_2_FC = 4.2). Another key functional category enriched in the L1 plants was “response to biotic stimulus”, including genes encoding pathogenesis-related (PR) proteins, phenylalanine ammonia lyases, stilbene synthases and trans-resveratrol di-*O*-methyltransferases, which are known to be associated with biotic stress responses in grapevine (Figure 7a; Supplementary Dataset S6) [6,31].

Most of these genes were commonly found in the “secondary metabolic process” category, together with genes encoding laccases, endochitinases, glutathion-S-transferases, peroxidases and gdsl esterase lipases, all families well known for their role in plant defense mechanisms (Figure 7a; Supplementary Dataset S6). Only a few functional categories were enriched among the 924 downregulated genes in L1 plants, and the statistical significance of enrichment was less striking than the upregulated genes (Figure 7b; Supplementary Dataset S7). The most notable categories were “lipid metabolic process”, including 15 genes encoding GDSL motif-containing lipase/esterase proteins, “secondary metabolic process” (mainly laccase genes), and “response to endogenous stimulus”, one third of which were auxin-related genes, supporting the proposed role of auxin in the susceptibility of plants to biotrophic pathogens [32], as well as several ethylene-responsive transcription factors. The few genes modulated in L1 plants following infection (L1i:L1m) were not enriched for any particular relevant functional category (Supplementary Figure S6). *VriATL156* expression therefore triggers the constitutive upregulation of genes primarily involved cell signaling (e.g., receptor-like kinases) and defense responses (pathogenesis-related proteins, enzymes involved in secondary metabolism), indicating the establishment of a pre-infection resistance status in L1 plants correlating with the observed resistance phenotype.

**Figure 8:**
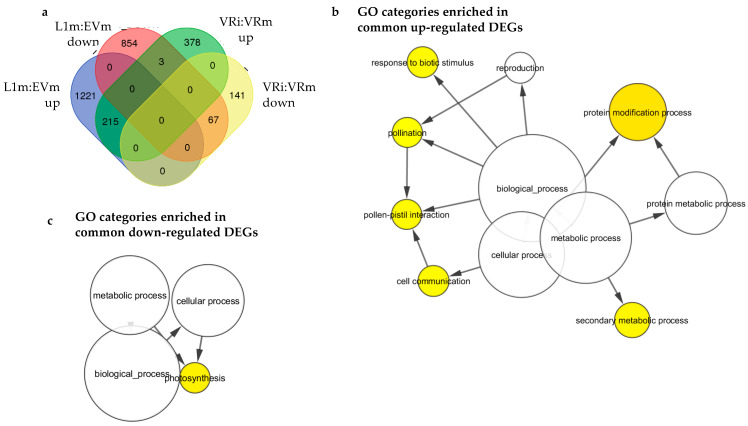
Comparison of differentially expressed genes at the basal level in mock-infected transgenic plants expressing VviATL156 (L1m) compared to empty vector controls (EVm) and genes that are modulated in *Vitis riparia* following infection with *P. viticola* (VRi vs. VRm). (**a**) Venn diagram showing differentially expressed genes between mock-infected transgenic and control plants (L1m vs. EVm) and infected vs. non-infected *Vitis riparia* (VRi vs. VRm) from Polesani et al. (2010). (**b**) BINGO enrichment analysis of upregulated genes in the above comparisons. (**c**) BINGO enrichment analysis of downregulated genes in the above comparisons. The size of the nodes is proportional to the number of genes in the test set which are annotated to that node, while the color of the node represents the corrected p-value. The node color scale ranges from white (not significantly over-represented) to yellow (*p*-value < 0.05).

The strong pre-infection expression level of many defense-related genes in L1 plants is reminiscent of the previously reported transcriptional comparison of *V. vinifera* and *V. riparia*, where all transcripts modulated in both species following infection with *P. viticola* were consistently more strongly regulated in *V. riparia* [16]. Accordingly, we compared the differentially expressed genes in non-infected L1 plants (this report) and in *V. riparia* plants infected with *P. viticola* [17], revealing that ~35% (282 out of 804) of genes modulated in infected *V. riparia* plants are similarly modulated in uninfected L1 plants, with only three genes showing an opposite regulation (i.e., upregulated in *V. riparia* while downregulated in the uninfected L1 line) (Figure 8a; Supplementary Figure S7; Supplementary Dataset S8). Moreover, this subset of common differentially expressed genes was enriched in all the functional categories representing the L1 transcriptional profile discussed above (Figure 8b,c; Supplementary Datasets S9 and S10). Interestingly, four ATL proteins (namely VviATL27, VviATL54b, VviATL149 and VviATL156) are commonly upregulated in both infected *V. riparia* and uninfected L1 plants (Supplementary Dataset S8). On the other hand, VviATL148 is upregulated only in the wild resistant *V. riparia*, while VvATL98 and VviATL110 are induced only in the L1 line, which also shows the specific downregulation of VviATL145 and VviATL86. This confirms the important co-regulation of grapevine ATLs and their responsiveness to biotic stress conditions [20,22]. Moreover, in line with the role of calcium in mediating defense signal transduction and the particular role of calcium-binding proteins in response to pathogens in grapevine [33], several grapevine calmodulin-like proteins (CMLs) are commonly or specifically regulated in *V. riparia* and L1 plants, thus correlating with the increased plant resistance of both genotypes compared to wild-type *V. vinifera*. These data suggest that *VriATL156* expression controlled by the constitutive CaMV 35S promoter mimics the effect of an infection response in the downy mildew-resistant grapevine species *V. riparia*, providing strong evidence that ATL156 is a key component in the defense response typical of resistant grapevine genotypes.

## 3. Discussion

Genetic resistance to disease is a highly valued grapevine trait because it avoids the need for chemical treatments, particularly the multiple chemical sprays required to control *P. viticola*. Genetic engineering allows the rapid transfer of such resistance traits to elite varieties without affecting their complex genetic background. Candidate resistance genes must be evaluated carefully, given the long and challenging process of grapevine transformation and regeneration. Several categories of genes can be considered, representing different layers of resistance: genes encoding receptors or signaling factors that regulate downstream defense responses, and genes encoding the components of those defense mechanisms [34]. We targeted the signaling layer, bypassing the specific pathogen recognition step in an attempt to engineer more durable resistance by preventing the adaptation of pathogens to individual receptors [12,35].

The candidate gene *VriATL156* from *V. riparia* is also present in *V. vinifera,* with minor sequence differences, i.e., the change from polar (Thr) to non-polar (Pro), predominantly affecting the protein C-terminus, which controls substrate recognition by E3 ubiquitin ligases and may therefore specify downstream ubiquitinated targets [36]. Following the recent release of the *V. riparia* genome [37], the cloned sequences were also compared with those predicted from the genome assembly, retrieving an additional SNP at the 3′ terminus, leading to an Arg to Gln amino acid substitution at position 316 of the corresponding proteins (Supplementary Figure S7), which would further support the observed differences between ATL156 from *V. vinifera* and *V. riparia* in the C terminal domain involved in substrate recognition. The overexpression of *VriATL156* in *V. vinifera* driven by the CaMV 35S promoter increased resistance to *P. viticola*, most effectively in line L1 and to a lesser extent in line L14, correlating with the transgene expression levels. This indicates a key role for this E3 ligase in disease resistance, in line with previous work on other ATL genes and co-expression network analysis [20,22,23]. RNA-Seq analysis showed that line L1 underwent broad transcriptional reprogramming relative to the control line, particularly involving genes in functional categories related to signal transduction and defense. These results indicate that defense mechanisms are already active at a basal level in plants overexpressing *VriATL156* and are not induced further by infection. Indeed, based on the functional analysis of the *VriATL156* and *VviATL156* promoters in the same *A. thaliana* genetic background, it is tempting to speculate that the higher basal activity of the *V. vinifera* promoter may not be sufficient to provide an effective response in the absence of strong post-infection induction, as observed in *V. riparia*. In contrast, the very high basal expression of *VriATL156* driven by the CaMV 35S promoter in line L1 may be sufficient to reach the threshold required to achieve resistance in *V. riparia* following infection. All genes induced in common between uninfected L1 plants and infected controls were expressed at a much higher level in the L1 plants. An interesting parallel can be drawn with the previous report comparing gene expression in *V. vinifera* and *V. riparia*, showing that all genes commonly modulated in these species in response to infection were induced more strongly in *V. riparia* [17]. This suggests that infection triggers a powerful amplification of the response in resistant species.

Line L1 also differed from the control in terms of hormonal signaling, with higher levels of salicylic and jasmonic acids in the uninfected L1 plants, in parallel with the downregulation of a number of auxin-related genes. The suppression of auxin-related genes is evidence that the hormone balance tips in favor of resistance in response to the transcriptional reprogramming, based on previous reports that auxins antagonize salicylic acid responses to favor growth and promote developmental processes, associated with susceptibility to biotrophic pathogens [33].

The molecular basis of the improved resistance in the transgenic lines expressing *VriATL156* is not clear because the upstream regulators and downstream targets have not been identified, as for other ATL E3 ubiquitin ligases including AtATL2 [38,39]. We previously reported that VviATL156 is co-regulated with several WRKY transcription factors across a number of plant–pathogen interactions, suggesting that one or more of these WRKY proteins may be regulators or targets of ATL156 [22]. Likewise, the E3 ubiquitin ligase EIRP1 targets the negative regulator of resistance VpWRKY11 in *Vitis pseudoreticulata* [40], and the E3 ubiquitin ligase ATL31 in *A. thaliana* is transcriptionally regulated by WRKY33 [41]. Additional evidence of ATL protein interactors includes EDR1 kinase, which negatively regulates AtATL1 to control programmed cell death and disease resistance [42], and the 14-3-3 protein targeted for degradation by ATL31 [43]. ATL31 also interacts with the SNARE protein SYP121 (involved in papilla formation), conferring resistance against powdery mildew in *A. thaliana* [43]. However, ATL31 does not seem to target SYP121 for degradation, probably acting as a regulator via other mechanisms [44].

The ability of ATL proteins to regulate their targets via mechanisms other than protein degradation reflects the well-established diverse effects of different modes of ubiquitination (mono-ubiquitination vs. poly-ubiquitination), different types of ubiquitin chain linkages, and different ubiquitin chain lengths. These assorted ubiquitin chains perform a range of functions, including the regulation of iron uptake, the repair of double-stranded breaks in DNA, and the regulation of auxin responses [45]. However, these diverse roles are poorly understood in plants [46,47]. Because the transgenic plants expressing *VriATL156* accumulate higher levels of ubiquitinated proteins, despite the absence of treatment with a proteasome inhibitor, the proteins ubiquitinated by VriATL156 may not be targeted for degradation. Resistance may not reflect the ubiquitin-proteasome system (UPS)-mediated degradation of particular negative defense regulators, but an unknown signaling role mediated by ubiquitin attachment, generating more canonical post-translational modifications [45].

To gain more insight into the function of VriATL156, we compared the transcriptomes of line L1 and control plants. The overexpression of VriATL156 affected thousands of transcripts, resulting in profound transcriptional reprogramming that redirected the transcriptome to activate defense-related signaling. Functional category enrichment revealed that these plants appear to be primed for defense, especially at the level of “protein modification”, with the upregulation of many genes encoding receptor kinases and putative E3 ubiquitin ligases, including ATLs. Some of these effects may reflect the constitutive expression of VriATL156 driven by the CaMV 35S promoter. However, the genes encoding defense effectors such as PR proteins and stilbene synthases, which are typically associated with disease resistance in grapevine, though enriched were less represented among the modulated transcripts compared with signaling components. This correlates with the lack of adverse phenotypic effects often observed when plants overexpress constitutive defenses, reflecting the damage incurred by plant tissues, for example, due to prolonged exposure to ROS [48].

In conclusion, our functional analysis has shown that *VriATL156* can regulate the resistance response in grapevine, and that the overexpression of this gene using a constitutive promoter leads to the strong pre-infection modulation of multiple components of the resistance signaling cascade. Given the vast transcriptomic reprogramming of resistance signaling components in the transgenic plants, this kind of resistance may be more durable than that achieved by expressing classical *R* genes because pathogens do not have the opportunity to evade specific receptors. The comparison of ATL156 native promoters in two grapevine species also provides insight into the contribution of VriALT156 in the resistant species *V. riparia*, which could facilitate the identification of pathogen-responsive motifs suitable for cisgenic strategies or genome editing approaches.

## 4. Materials and Methods

### 4.1. Bioinformatics

The VviATL156 coding sequence was retrieved from the published genome of *V. vinifera* cv. Pinot Noir genome [2]. (http://genomes.cribi.unipd.it/grape/). The coding sequence for *VriATL156* was determined by amplifying the genomic DNA from *V. riparia* cv. Gloire de Montpellier using primers based on the *V. vinifera* sequence because the *V. riparia* genome had not been sequenced at the time of our experiments. Putative transcriptional start sites (TSSs) and TATA boxes were predicted using TSSPlant (http://www.softberry.com), cis-acting elements were predicted using PlantCARE [24] (http://bioinformatics.psb.ugent.be/webtools/plantcare/html/) and transcription factor binding sites were identified using PlantPAN2.0 [49]; (http://plantpan2.itps.ncku.edu.tw/). Alignments were performed with Multalin [50].

### 4.2. Cloning the VriATL156 Coding Sequence

Genomic DNA extracted from the leaves of *V. riparia* Gloire de Montpellier plants [51] was amplified with High Fidelity DNA Polymerase (KAPA Biosystems, Roche, Basel, Switzerland) and the following primers, designed using Primer3 [52] and prepared by Eurofins Genomics (Milan, Italy): ATL2_int_For (5′-GAT CGA CTT CCG CTT TGT GCT CC-3′) and ATL2_int_Rev (5′-GCA CAC GGC ACA TTC AG AC-3′). The *VriATL156* gene was verified by sequencing (BMR Genomics, Padua, Italy) and transferred to a pGEM vector under the control of the CaMV 35S promoter for cloning in *Escherichia coli*. The cassette was then transferred to pART7 [28] and finally isolated as NotI fragment and inserted at the NotI site of the modified binary vector pART27-4a (also containing an eGFP5 expression cassette driven by the *A. thaliana* ubiquitin 10 promoter) for grapevine transformation. The vector (10 ng) was introduced into *A. tumefaciens* strain EHA105 by electroporation (25 μF, 2.5 KVolt, 2000 Ohm). Transformed cells were rapidly collected with 1 mL of Lysogeny broth (LB) medium and incubated for 1 h at 28° and 200 rpm. Then, 75-150-225 μL of cell suspension was inoculated into a solid selective medium and incubated at 28 °C for 48 h.

### 4.3. Cloning the VviATL156 and VriATL156 Promoter Regions

A putative promoter region extending ~1.2 kb upstream of the transcriptional start site was isolated from both genes using primers ATL156for (5′-**CAC C**GA CCT TTT ACT ATA CAT AGG TAG-3′) and ATL156rev (5′-**TT**T CTC CGA TCT GAA AGA GAT AAA G-3′), carrying the CACC and the TT clamps (bold italics) for directional cloning in pENTR™/D-TOPO^®^ (Invitrogen) and transformation into *E. coli* Top10 competent cells.

### 4.4. Analysis of Promoter Responses in Transgenic A. thaliana

The cloned promoter regions of *VviATL156* and *VriATL156* were transferred to vector pKGWFS7 and introduced into *A. tumefaciens* strain GV3101 for the transformation of *A. thaliana* Col-0 plants by floral dipping [53]. T3 transgenic lines carrying the *gusA* gene under the control of the *VriATL156* promoter (Vri lines 4 and 8) or the *VviATL156* promoter (Vvi lines 3 and 10) were grown in Murashige and Skoog (MS) medium from 3 days to 4 weeks with a 16-h photoperiod at 24 °C. The same lines were also cultivated in soil pots for 4 weeks for infection challenge experiments with *Pseudomonas syringae* pv. *tomato* (PstDC3000). Basal promoter activity was measured after 24, 48 and 96 h, and after 1, 2, 3 and 4 weeks. Three plants from each line were sampled at each time point for GUS staining and the measurement of GUS activity.

Chemical stimuli were applied to 5-day-old transgenic plants from the four lines described above as well as vector-only controls. The chemicals were applied to plants in 96-well plates containing half-strength liquid MS medium supplemented with methylsalicylic acid, methyljasmonate, abscisic acid or ethephon (ethylene donor), at indicated concentrations, and promoter activity was tested after 24 h. For the challenge studies, transgenic plants were infected with *Pseudomonas syringae* pv. *tomato* strains AvrB (avirulent on Col-0) or DC3000 (virulent) at 2 × 10^7^ CFU/mL, by the infiltration of three biological replicates using a 1 mL needless syringe. Mock inoculations were carried out with 10 mM MgCl_2_.

### 4.5. GUS Staining and GUS Fluorimetric Assay

Whole plants or detached leaves (*A. thaliana* lines Vri 4.17, Vri 8.4, Vvi 3.14 and Vvi 10.7) were submerged in GUS staining solution (2.5 mM Na_3_PO_4_, pH 7.0, 0.1% Triton x-100, 100 µM X-Gluc) in a vacuum chamber for 5 min and then incubated at 37 °C for 5 h before clearing in 70% ethanol and visualization with a LEICA MZ16F stereomicroscope. The GUS fluorimetric assay was carried out as previously described (DyNA Quant Application Note 3) using a pool of three leaves from each biological replicate. Fluorescence was measured using a 3300 Nanodrop fluorimeter (Thermo Fisher Scientific, Waltham, MA, USA). Proteins were quantified in 1 mL of extract using Bradford reagent (Bio-Rad Laboratories, Hercules, CA, USA).

### 4.6. Transformation of V. vinifera

Pre-embryogenic callus of *V. vinifera* cv Shiraz was generated from anthers collected at the pre-flowering stage and initiated on callus initiation medium (PIV) medium before transfer to charcoal containing medium (GS1CA) C1 medium [54]. Transformation was carried out as previously described [55,56]. Callus was selected on C1 medium supplemented with 150 μg/mL kanamycin, and stably transformed embryos were screened for the presence of GFP, transferred to shooting medium and then deflasked as previously described [57].

Transformation was confirmed by Southern blot analysis using a probe designed to specifically recognize a sequence encompassing the final portion of the ubiquitin terminator (GFP expression cassette) and a portion of the T-DNA region next to the left border. The probe was labeled with alkaline phosphatase using the AlkPhos Direct Kit (GE Healthcare, Amersham, UK).

Transgenic plants plus controls (regenerated Shiraz vines transformed with the empty vector pART27) were regularly micropropagated in vitro and cultivated in rooting medium in a growth chamber at 27 °C with a 16-h photoperiod. Rooted plantlets were transferred to soil and cultivated in the greenhouse under shade for ~3 weeks before returning to normal conditions. Plants were tested for resistance about 3 months after transfer to the soil.

### 4.7. Infection with P. viticola

An isolate of *P. viticola* harvested from a local field in the Verona province (Italy) in 2013 was propagated axenically as previously described [17]. For experimental infections, leaf disks were cut from the sterilized 3rd, 4th and 5th leaves from the apex of plant shoots and inoculated with 50 μL drops (30,000 sporangia/mL). Inoculated disks were kept in the dark overnight and inoculum droplets were removed after 24 h. Distilled water drops were used as control and collected at the same time points. Disks were incubated in a growth chamber with a 16-h photoperiod at 21–24 °C and were monitored for 6 days, until sporulation was observed on the controls. For microscopy, leaf disks were collected 24, 48 and 96 hpi, stained with 0.05% (*w*/*v*) aniline blue in 0.1% (*w*/*v*) Na_2_CO_3_ (pH 10) and observed under a Leica DM/RB epifluorescence microscope (Leica Microsystems, Wetzlar, Germany) with excitation band pass filter (BP) 340–380 nm, dichroic mirror 400 nm and suppression log pass filter (LP) > 430 nm. For RNA-Seq analysis, leaf disks were collected 24 hpi, immediately frozen in liquid nitrogen and stored at –80 °C. Three independent biological replicates were artificially infected for analysis.

### 4.8. Analysis of Endogenous Hormone Levels

We extracted 200 mg of powdered leaf tissue in 2 mL methanol-water (7:3 *v*/*v*) containing 1% acetic acid by sonication for 15 min at 4 °C. Extractions were performed from 3 independent samples. The samples were centrifuged at 16,000× *g* for 5 min at 4 °C and the pellet was re-extracted with the same solvent. The supernatants were pooled and evaporated in a speed-vac to a final volume of 100 µl before the analysis of 2 μL samples by HPLC-MS/MS on an Agilent 1100 HPLC system (Agilent Technologies, Santa Clara, CA, USA) connected to an LTQ Orbitrap mass spectrometer (Thermo Fisher Scientific) equipped with a Luna-HILIC column (100 mm × 2.0 mm, 5 μm column; Phenomenex, Torrance, CA, USA). The solvent gradient was 50% A (acetonitrile containing 1% formic acid) and 50% B (water containing 1% formic acid) to 100% B over 20 min at a flow rate of 1.5 mL/min. The injection volume was 2 µL.

The MS was operated in negative ion mode. Selected reaction monitoring (SRM) experiments were used to monitor specific precursor ion → product ion transitions for each phytohormone. Collision energy, precursor ion isolation width and activation Q were optimized for each compound separately. The following mass transitions were monitored: jasmonic acid 209 > (58–60), salicylic acid 137 > (92–94), ABA 263 > (152–154), and OPDA 291 > (164–166). Stock solutions of each phytohormone standard were prepared and working solutions were prepared, diluting these for the calibration curve. All solvents used for the extraction and LC-MS/MS were analytical grade or HPLC grade. Indole-3-acetic acid (purity > 99%), ABA (purity > 99%), salicylic acid (purity > 98%), and jasmonic acid (purity > 95%) were obtained from MilliporeSigma (Merck, Darmstadt, Germany) and OPDA was obtained from Cayman (Biomol, Hamburg, Germany).

### 4.9. Quantitative Real-Time PCR (qPCR)

Grapevine leaf disks (in biological triplicate) were ground in liquid nitrogen and total RNA was isolated from 200 mg of powder per sample, using the Spectrum Plant Total RNA Kit (MilliporeSigma). The quantity, integrity and purity of the RNA were determined using a Nanodrop 2000 (Thermo Fisher Scientific) and a Bioanalyzer Chip RNA 7500 series II (Agilent Technologies). The RNA was treated with DNase from the TURBO DNA-free kit (Thermo Fisher Scientific) and 1 µg was reverse transcribed using the SuperScript III kit (Invitrogen, Thermo Fisher Scientific). qPCR was carried out using the SYBR Green PCR Master Mix kit (Applied Biosystems, Waltham, MA, USA). The ubiquitin1-encoding gene (VIT_16s0098g01190) was used as the reference for expression normalization. The list of primers is provided in Supplementary Table S1. The “Comparative Quantitation” protocol was applied using the Mx3000P Real-time qPCR system (Stratagene, Agilent Technologies). The real amplification efficiency was calculated using LinRegPCR [58]. Mean normalized expression (MNE) values were calculated according to [59]. Standard errors (SE) were calculated from three biological replicates.

### 4.10. Western Blot

Leaves from transgenic lines L1 and L14 and control (regenerated empty vector-transformed) *V. vinifera* cv. Shiraz plants were ground in liquid nitrogen and protein was extracted from 100 mg of powder as previously described [60]. Samples (equivalent to 35 µg total protein) were fractionated by SDS-PAGE and transferred to a nitrocellulose membrane (GE Healthcare) at 100 V for 1 h. Membranes were incubated with a primary anti-ubiquitin antibody 1:2000 (Enzo Life Sciences, Inc.) and a secondary anti-mouse-HRP IgG antibody 1:50,000 in Tris buffer saline medium supplemented with 0.1% Tween 20 (TBS/T) with milk 5%, for 2 h and 1.5 h, respectively, at room temperature. Finally, bands were revealed by exposing membranes to chemiluminescent substrate (Amersham™ ECL Western Blotting Detection Kit, GE Healthcare). Images were acquired with a ChemiDoc imaging system (Bio-Rad).

### 4.11. RNA-Seq Analysis

For each sample, an unstranded library was prepared from 2.5 µg of total RNA using the Illumina TruSeq Library Prep Kit v2 (Illumina, San Diego, CA, USA) and was sequenced on an Illumina HiSeq500 (101-bp, paired-end reads) at the Functional Genomics Center (Department of Biotechnology, University of Verona, Italy). The data were preprocessed and analyzed using high performance computing resources made available by CINECA (Class C ISCRA project IsC33_NobleRot). Raw reads were pre-processed using trimmomatic [61] to remove low-quality bases and adapter sequences. Filtered reads were mapped against the reference grapevine genome [2] using TopHat v2.0.11 [62]. Mapped reads were summarized at the gene level into a count-matrix using the htseq-count tool from the HTSeq library [63]. Gene models provided to the htseq-count tool to score reads mapping unambiguously to a single gene were obtained from the grape genome database release V1 (http://genomes.cribi.unipd.it/DATA/) [64]. Differentially expressed genes were identified and analyzed using EdgeR software [65]. The raw read counts were normalized to the gene GC content using the EDASeq package [66] and sequencing depth among samples (TMM trimmed mean of M values normalization in EdgeR). We considered as differentially expressed all the genes with a p value < 0.05 after false discovery rate correction and a Log2 fold change >|1|.

## Data Availability

RNA-seq data are available as Supplementary Data Matrix.

## Supplementary Materials

The following are available online at https://www.mdpi.com/1422-0067/22/2/940/s1.
