# Supplementary material for: The Grapevine E3 Ubiquitin Ligase VriATL156 Confers Resistance against the Downy Mildew Pathogen Plasmopara viticola
Source: Int J Mol Sci. 2021 Jan 19;22(2):940. doi: 10.3390/ijms22020940 (PMC7833427; doi:10.3390/ijms22020940)
Supplement: Supplementary file 1 [file ijms-22-00940-s001.zip › SUPPLEMENTARY FILES/Supplementary Figures S1-S7+original western blot.pdf]

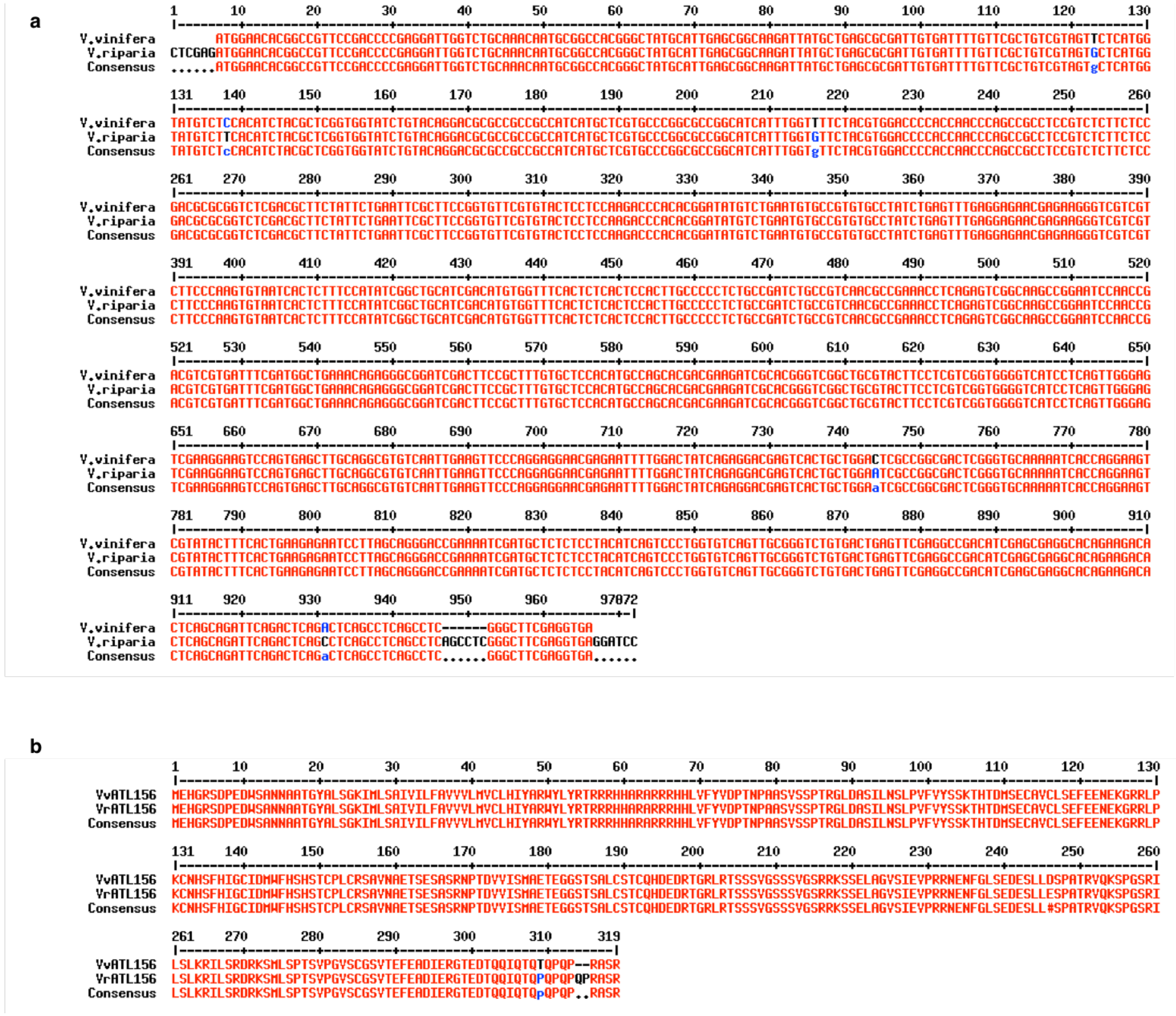
a
V.vinifera
V.riparia
Consensus
b
VvATL156
VrATL156
Consensus

Figure S1

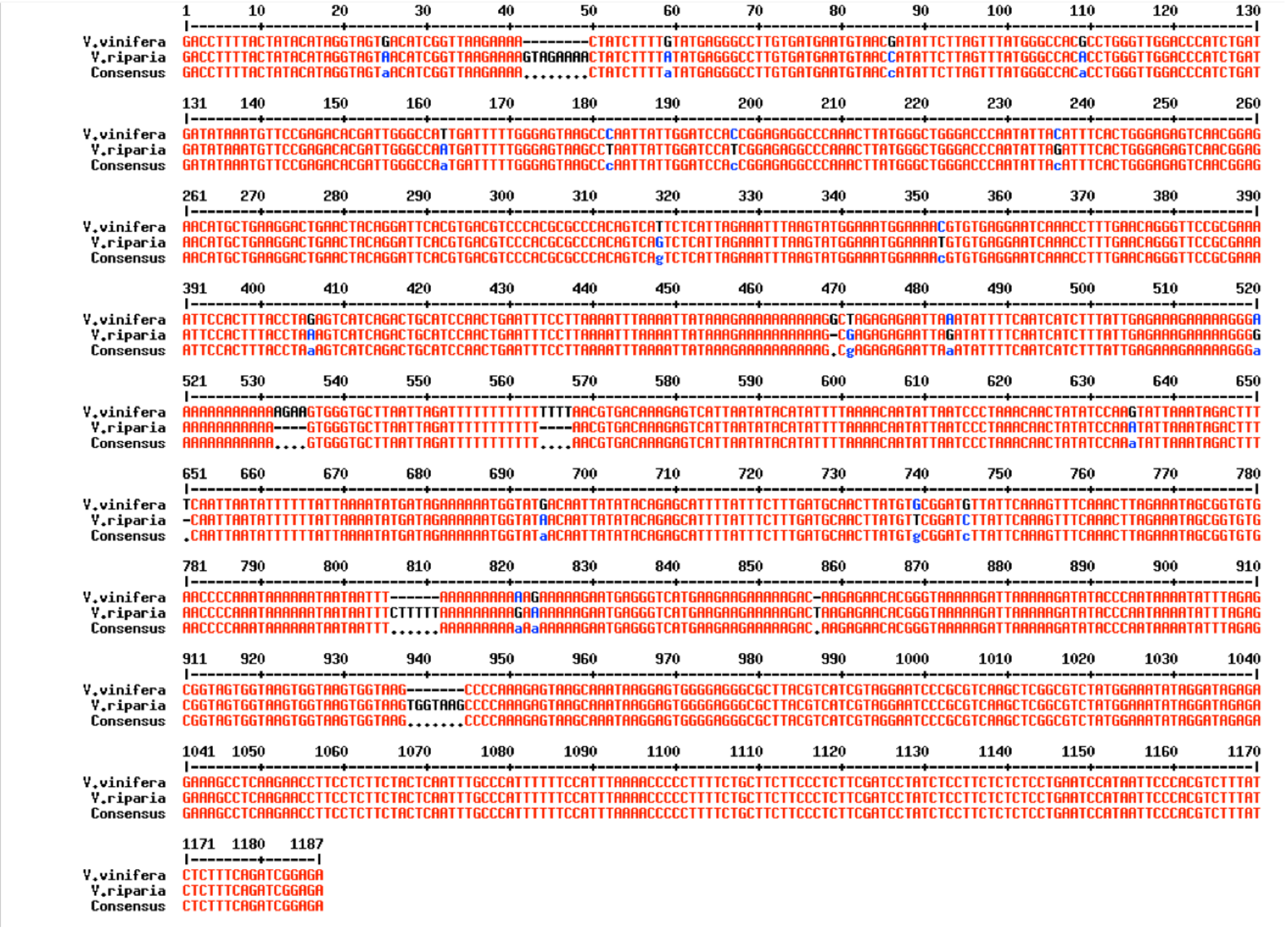

Figure S2

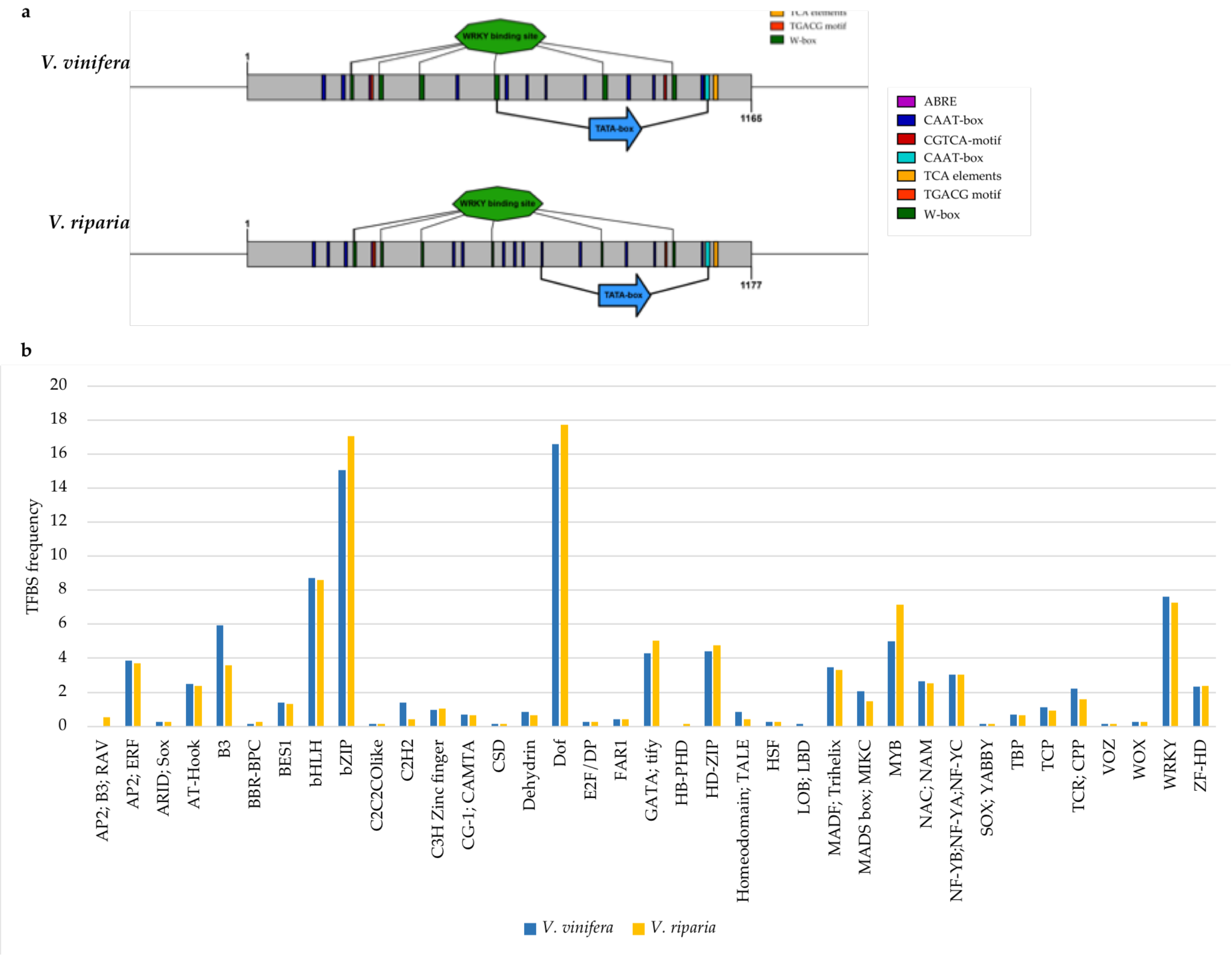
a
V. vinifera
V. riparia
WRKY binding site
TATA-box
1
1165
1177
ABRE
CAAT-box
CGTCA-motif
CAAT-box
TCA elements
TGACG motif
W-box
b
TFBS frequency
0
2
4
6
8
10
12
14
16
18
20
AP2; B3; RAV
AP2; ERF
ARID; Sox
AT-Hook
B3
BBR-BPC
BES1
bHLH
bZIP
C2C2COlike
C2H2
C3H Zinc finger
CG-1; CAMTA
CSD
Dehydrin
Dof
E2F/DP
FAR1
GATA; tify
HB-PHD
HD-ZIP
Homeodomain; TALE
HSF
LOB; LBD
MADF; Trihelix
MADS box; MIKC
MYB
NAC; NAM
NF-YB;NF-YA;NF-YC
SOX; YABBY
TBP
TCP
TCR; CPP
VOZ
WOX
WRKY
ZF-HD
V. vinifera
V. riparia

Figure S3

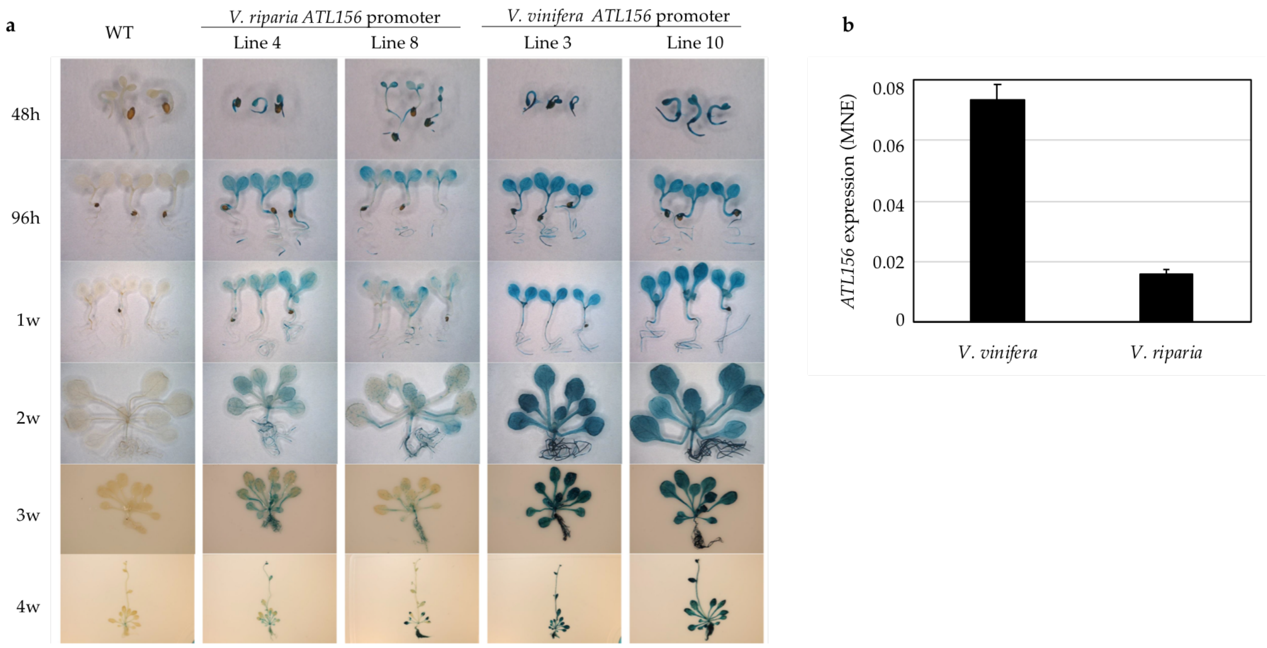
a
WT
V. riparia ATL156 promoter
Line 4
Line 8
V. vinifera ATL156 promoter
Line 3
Line 10
48h
96h
1w
2w
3w
4w
b
ATL156 expression (MNE)
0.08
0.06
0.04
0.02
0
V. vinifera
V. riparia

Figure S4

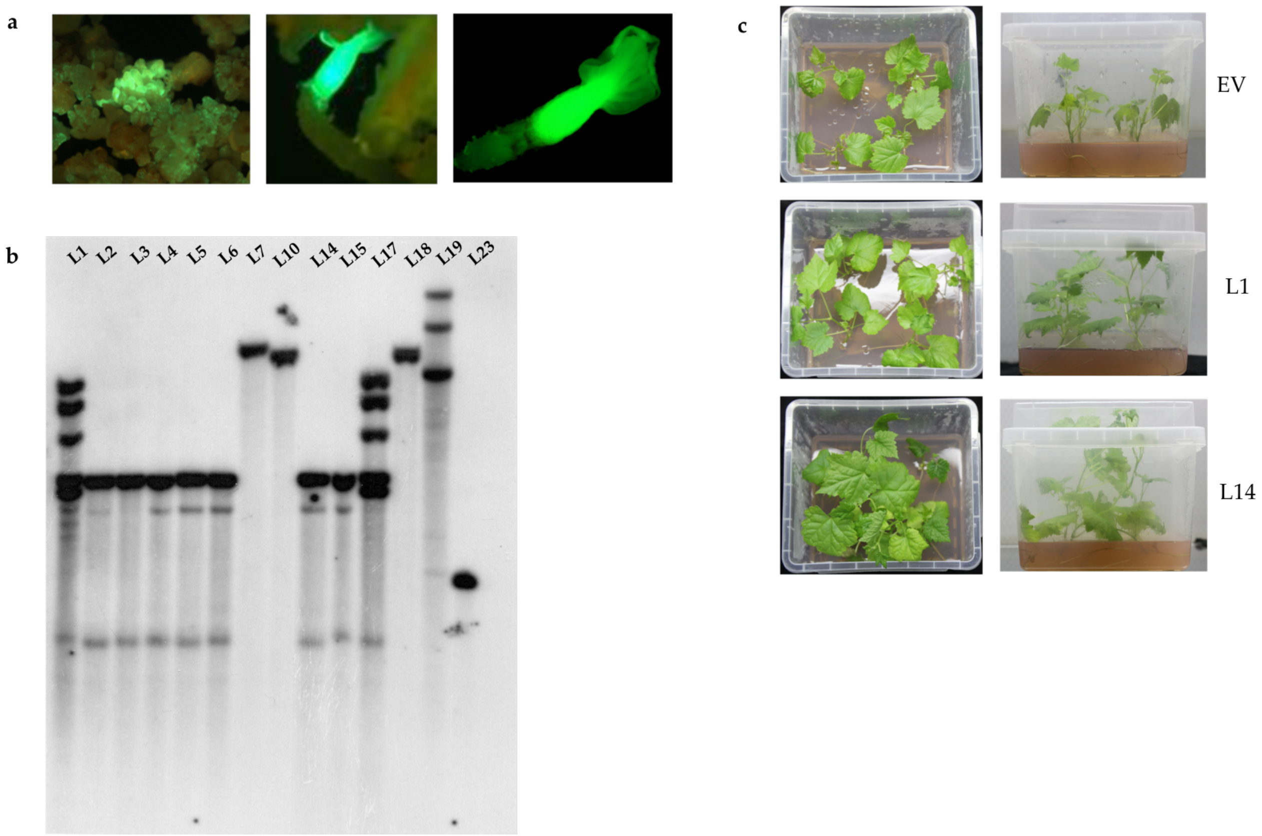

Figure S5

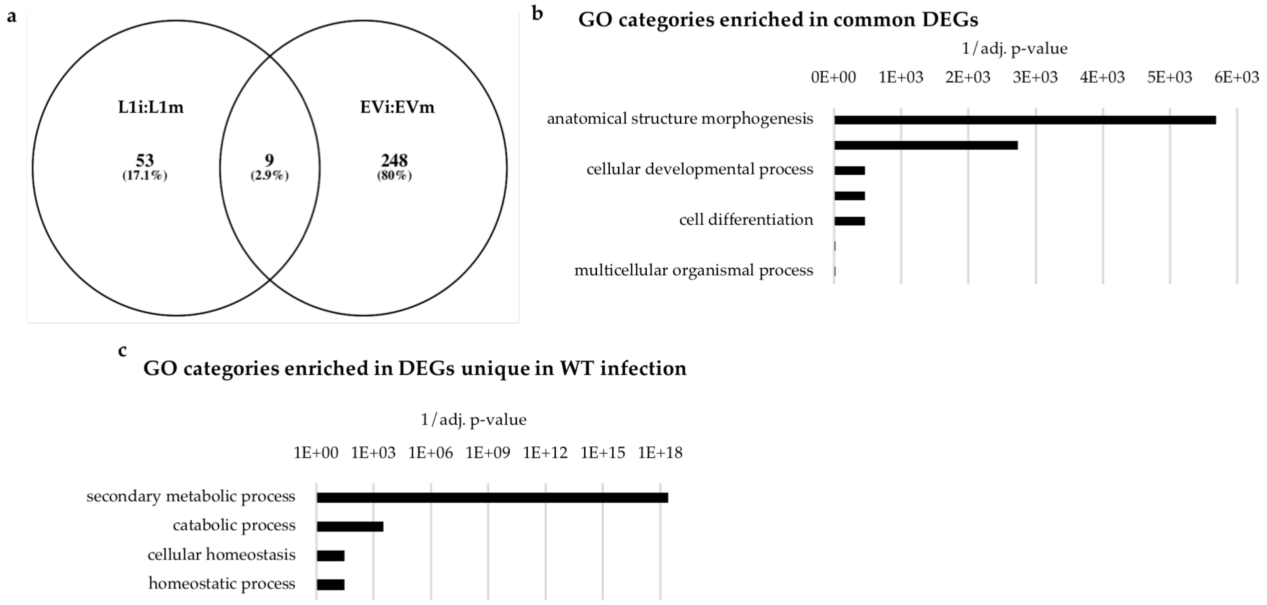
a
L1i:L1m
53
(17.1%)
9
(2.9%)
EVi:EVm
248
(80%)
b
GO categories enriched in common DEGs
1/adj. p-value
0E+00 1E+03 2E+03 3E+03 4E+03 5E+03 6E+03
anatomical structure morphogenesis
cellular developmental process
cell differentiation
multicellular organismal process
c
GO categories enriched in DEGs unique in WT infection
1/adj. p-value
1E+00 1E+03 1E+06 1E+09 1E+12 1E+15 1E+18
secondary metabolic process
catabolic process
cellular homeostasis
homeostatic process

Figure S6

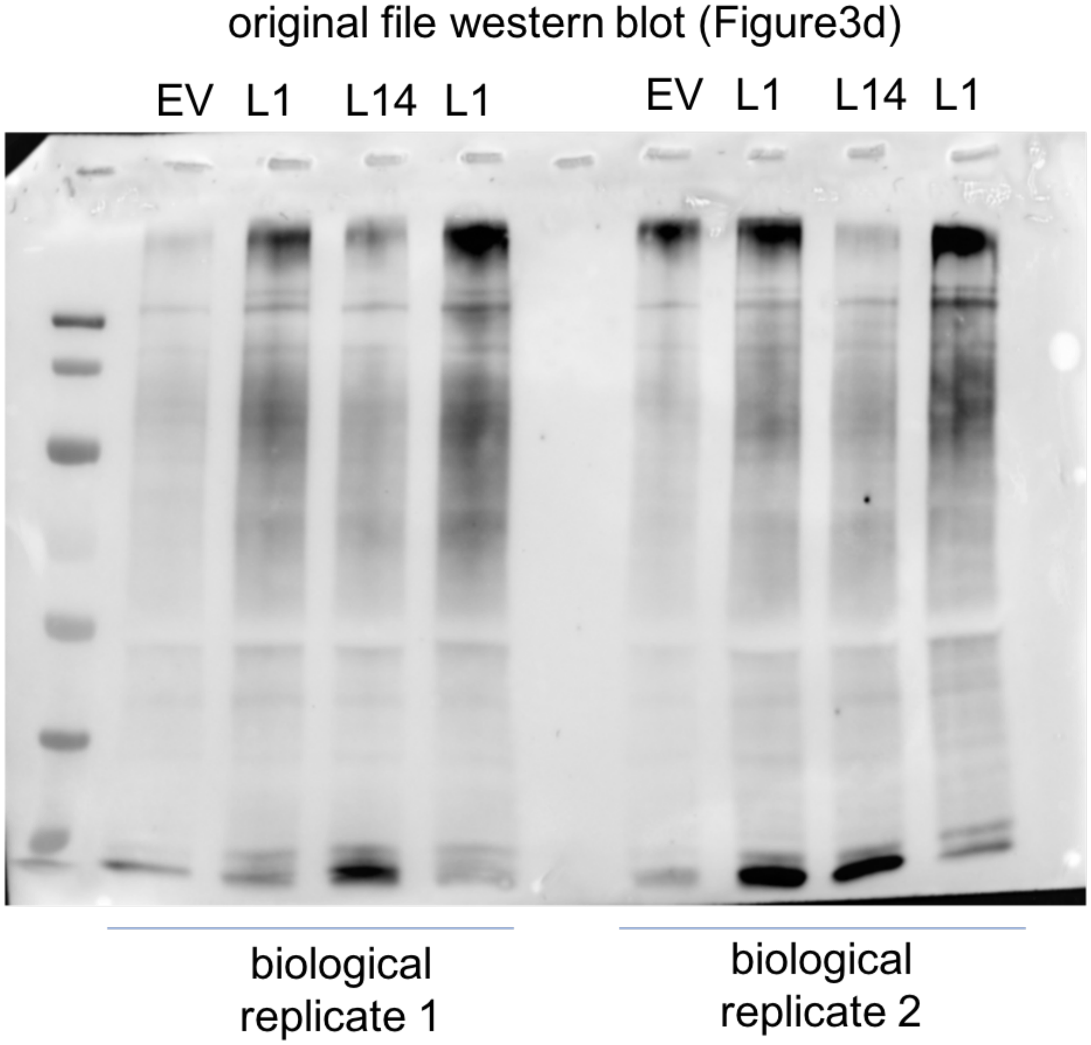

Original File (uncropped) western blot anti-Ub (Figure 3d)

```
              1        10        20        30        40        50        60        70        80        90        100       110       120       130
              |--------+---------+---------+---------+---------+---------+---------+---------+---------+---------+---------+---------+---------|
   VviATL156  MEHGRSDPEDWSANNAATGYALSGKIMLSAIVILFAVVVLMVCLHIYARWYLYRTRRRHHARARRRHHLVFYVDPTNPAASVSSPTRGLDASILNSLPVFVYSSKTHTDMSECAVCLSEFEENEKGRRLP
   VriATL156  MEHGRSDPEDWSANNAATGYALSGKIMLSAIVILFAVVVLMVCLHIYARWYLYRTRRRHHARARRRHHLVFYVDPTNPAASVSSPTRGLDASILNSLPVFVYSSKTHTDMSECAVCLSEFEENEKGRRLP
vitri05g00300  MEHGRSDPEDWSANNAATGYALSGKIMLSAIVILFAVVVLMVCLHIYARWYLYRTRRRHHARARRRHHLVFYVDPTNPAASVSSPTRGLDASILNSLPVFVYSSKTHTDMSECAVCLSEFEENEKGRRLP
   Consensus  MEHGRSDPEDWSANNAATGYALSGKIMLSAIVILFAVVVLMVCLHIYARWYLYRTRRRHHARARRRHHLVFYVDPTNPAASVSSPTRGLDASILNSLPVFVYSSKTHTDMSECAVCLSEFEENEKGRRLP

              131      140       150       160       170       180       190       200       210       220       230       240       250       260
              |--------+---------+---------+---------+---------+---------+---------+---------+---------+---------+---------+---------+---------|
   VviATL156  KCNHSFHIGCIDMWFHSHSTCPLCRSAVNAETSESASRNPTDVVISMAETEGGSTSALCSTCQHDEDRTGRLRTSSSVGSSSVGSRRKSSELAGVSIEVPRRNENFGLSEDESLLDSPATRVQKSPGSRI
   VriATL156  KCNHSFHIGCIDMWFHSHSTCPLCRSAVNAETSESASRNPTDVVISMAETEGGSTSALCSTCQHDEDRTGRLRTSSSVGSSSVGSRRKSSELAGVSIEVPRRNENFGLSEDESLLESPATRVQKSPGSRI
vitri05g00300  KCNHSFHIGCIDMWFHSHSTCPLCRSAVNAETSESASRNPTDVVISMAETEGGSTSALCSTCQHDEDRTGRLRTSSSVGSSSVGSRRKSSELAGVSIEVPRRNENFGLSEDESLLESPATRVQKSPGSRI
   Consensus  KCNHSFHIGCIDMWFHSHSTCPLCRSAVNAETSESASRNPTDVVISMAETEGGSTSALCSTCQHDEDRTGRLRTSSSVGSSSVGSRRKSSELAGVSIEVPRRNENFGLSEDESLL#SPATRVQKSPGSRI

              261      270       280       290       300       310      319
              |--------+---------+---------+---------+---------+--------|
   VviATL156  LSLKRILSRDRKSMLSPTSVPGVSCGSVTEFEADIERGTEDTQQIQTQTQPQP--RASR
   VriATL156  LSLKRILSRDRKSMLSPTSVPGVSCGSVTEFEADIERGTEDTQQIQTQPQPQPQPRASR
vitri05g00300  LSLKRILSRDRKSMLSPTSVPGVSCGSVTEFEADIERGTEDTQQIQTQPQPQPQPQASR
   Consensus  LSLKRILSRDRKSMLSPTSVPGVSCGSVTEFEADIERGTEDTQQIQTQpQPQPqprASR
```

Figure S7
