# Supplementary material for: The Grapevine E3 Ubiquitin Ligase VriATL156 Confers Resistance against the Downy Mildew Pathogen Plasmopara viticola
Source: Int J Mol Sci. 2021 Jan 19;22(2):940. doi: 10.3390/ijms22020940 (PMC7833427; doi:10.3390/ijms22020940)
Supplement: Supplementary file 1 [file ijms-22-00940-s001.zip › SUPPLEMENTARY FILES/Supplementary Table 1.pdf]

Supplementary Table 1. List of primers used for qPCR analysis.

Primer Name	Primer sequences	Tm (°C)	Amplicon size
ATL156_for	TGAGTTTGAGGAGAACGAGAAGG	60.6	100 bp
ATL156_rev	GCAAGTGGAGTGAGAGTGAAACC	62.4	
UBI_for	GCCCTGCACTTACCATCTTTAAG	60.6	100 bp
UBI_rev	GAGGGTCGTCAGGATTTGGA	59.4	
